# Event-related brain potential correlates of emotional face processing

**DOI:** 10.1016/j.neuropsychologia.2006.04.022

**Published:** 2007

**Authors:** Martin Eimer, Amanda Holmes

**Affiliations:** a Department of Psychology, Birkbeck College, University of London, Malet Street, London WC1E 7HX, UK; b School of Human and Life Sciences, Roehampton University, London, UK

**Keywords:** Face processing, Emotion, Emotional facial expression, Event-related brain potentials, Cognitive affective neuroscience

## Abstract

Results from recent event-related brain potential (ERP) studies investigating brain processes involved in the detection and analysis of emotional facial expression are reviewed. In all experiments, emotional faces were found to trigger an increased ERP positivity relative to neutral faces. The onset of this emotional expression effect was remarkably early, ranging from 120 to 180 ms post-stimulus in different experiments where faces were either presented at fixation or laterally, and with or without non-face distractor stimuli. While broadly distributed positive deflections beyond 250 ms post-stimulus have been found in previous studies for non-face stimuli, the early frontocentrally distributed phase of this emotional positivity is most likely face-specific. Similar emotional expression effects were found for six basic emotions, suggesting that these effects are not primarily generated within neural structures specialised for the automatic detection of specific emotions. Expression effects were eliminated when attention was directed away from the location of peripherally presented emotional faces, indicating that they are not linked to pre-attentive emotional processing. When foveal faces were unattended, expression effects were attenuated, but not completely eliminated. It is suggested that these ERP correlates of emotional face processing reflect activity within a neocortical system where representations of emotional content are generated in a task-dependent fashion for the adaptive intentional control of behaviour. Given the early onset of the emotion-specific effects reviewed here, it is likely that this system is activated in parallel with the ongoing evaluation of emotional content in the amygdala and related subcortical brain circuits.

## 1 Introduction

The investigation of emotional states, their neural correlates, and their role for the regulation of cognition and action is now one of the most active research areas in cognitive neuroscience (see Adolphs, 2003; Dolan, 2002, for reviews). On the most general level, emotional states are evolutionary adaptations that are critically involved in the regulation of basic survival mechanisms, and in the control of behaviour in complex environments (Damasio, 1999). A number of complementary methods, such as single cell recordings, functional brain imaging, or neuropsychological investigations of focal brain damage have been used to identify brain structures that are involved in the perception and analysis of emotionally significant information, mediate bodily emotional responses, and control social cognition and behaviour.

Many recent studies have investigated the neural network underlying emotional processing by measuring brain responses to emotionally salient stimuli. Differences in brain responses to stimuli that vary in their emotional content have been interpreted as evidence for functional specialisation among neural processes responsible for the processing of emotional information. Such studies have revealed a complex interconnected network of brain structures responsible for the analysis of emotional events. This network includes higher order sensory cortices, where perceptual representations of emotionally relevant stimuli are formed, and the amygdala, orbitofrontal cortex, and ventral striatum, where such sensory representations appear to be classified in terms of their emotional significance. It also includes paralimbic and higher cortical areas such as somatosensory cortex, anterior cingulate, and medial prefrontal cortex, where conscious representations of emotional states are generated, which can be used in the strategic control of behaviour in complex social situations, and in the planning of future goals and actions (see Adolphs, 2003, for more details).

Emotional facial expressions are particularly salient stimuli for conveying important nonverbal communications to other species members, and, in humans, are immediate indicators of affective dispositions in other people. Because of this paramount emotional significance of facial expression, numerous recent functional imaging, lesion, and single-cell recording studies have used emotional faces to identify neural substrates of emotional processing. These studies have found that brain areas generally involved in the processing of emotional information (see above) are also activated during the processing of facial emotion. The initial perceptual analysis of faces takes place in inferior occipital cortex (‘occipital face area’; see Rossion et al., 2003) and in the middle fusiform gyrus for structural properties of faces which determine face identity (Hoffman & Haxby, 2000; for reviews, see Haxby, Hoffman, & Gobbini, 2000; Gobbini & Haxby, 2006, this issue). The superior temporal sulcus is involved in the processing of dynamic aspects of faces, such as facial expression, eye and mouth movements (Allison, Puce, & McCarthy, 2000; see also Puce, Epling, Thompson, & Carrick, 2006, this issue). A rapid evaluation of the emotional and motivational significance of facial expression appears to be mediated by the amygdala and orbitofrontal cortex, while structures such as the anterior cingulate, prefrontal cortex and somatosensory areas are linked to the conscious representation of emotional facial expression for the strategic control of thought and action, as well as to the production of concomitant feeling states (see Adolphs, 2003, for more details).

In summary, recent neuroscientific investigations of emotional processing have uncovered components of a complex network for the detection and analysis of emotionally significant information. Since most of these recent studies have used fMRI measures, which are based on relatively slow hemodynamic brain responses to emotional stimuli, information about the time course of emotional processing has been relatively scarce. The availability of detailed temporal information is necessary to obtain a more comprehensive picture of the functional properties of the emotional brain. Thus, fMRI measures need to be complemented with methods that provide insights into temporal parameters of emotional processing, such as event-related brain potential (ERP) or magnetoencephalographic (MEG) measures.

This paper reviews a series of recent studies in our lab that have used ERP measures to investigate the processes involved in the detection and analysis of emotional facial expression. In Section 2, we will introduce ERP correlates of emotional face processing by discussing differential ERP responses to fearful versus neutral faces. Section 3 will review ERP modulations triggered by other basic emotional expressions. In Section 4, we will present ERP results that support the hypothesis that the structural encoding of faces and the detection of their emotional expression represent parallel and independent processes. Section 5 will discuss the impact of selective attention on ERP responses elicited by emotional facial expression, and Section 6 will briefly review recent findings concerning the impact of the spatial frequency content on such emotional expression effects. Overall, the studies reviewed in this paper will demonstrate that ERPs represent a useful tool to study the time course and the functional properties of emotional face processing stages, such as their automaticity, specificity, and sensitivity to attentional states. Perhaps most importantly, our studies have shown that although selective brain responses to emotional faces, as measured with ERPs, are triggered at very short latencies, they are strongly dependent on attention. This suggests that they are not directly linked to the initial automatic detection of emotional content mediated by the amygdala and related structures, but rather to subsequent, cognitively penetrable stages of emotional processing.

## 2 ERP correlates of emotional face processing: fearful faces

The basic question addressed in our initial study (Eimer & Holmes, 2002) was when and how the difference between emotional and neutral facial expressions would be reflected in ERP waveforms. We recorded ERPs while participants viewed photographs of single fearful faces, neutral faces, or houses, which were presented at the centre of a computer screen. As in all other studies reported below, fearful and neutral faces were taken from a standard set of pictures of facial affect (Ekman & Friesen, 1976). Participants’ task was to detect infrequent immediate repetitions of identical stimuli across successive trials. Half of all fearful and neutral faces were presented in their standard upright orientation, while the other half was presented upside-down. Because face inversion disrupts not only face identification, but also to some degree the recognition of emotional facial expression (de Gelder, Teunisse, & Benson, 1997; Searcy & Bartlett, 1996), we expected ERP modulations indicative of the detection and processing of facial expression to be attenuated and possibly also delayed in response to inverted relative to upright faces.

**Fig. 1:**
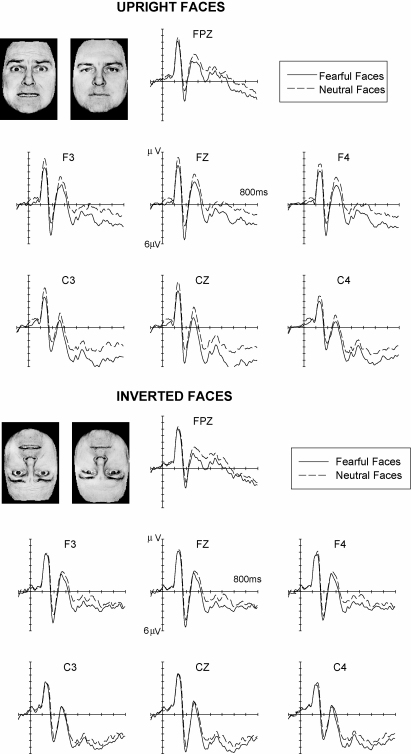
Grand-averaged ERP waveforms in response to fearful faces (solid lines) and neutral faces (dashed lines), displayed separately for upright faces (top panel) and inverted faces (bottom panel). Data from Eimer and Holmes (2002).

**Fig. 2:**
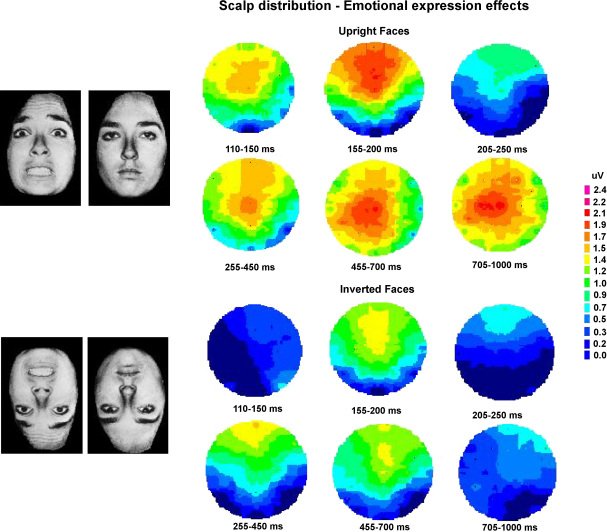
Topographical maps showing scalp distributions of emotional expression effects for upright faces (top) and inverted faces (bottom), obtained by subtracting ERPs to neutral faces from ERPs to fearful faces within six successive post-stimulus latency windows. Red colours indicate an enhanced positivity for fearful relative to neutral faces, while blue colours indicate small or absent amplitude differences. Data from Eimer and Holmes (2002).

Fig. 1 shows ERPs triggered in response to fearful faces and neutral faces, for faces presented in their usual upright orientation (top panel), and for inverted faces (bottom panel). An enhanced positivity was present for ERPs to fearful relative to neutral faces, and this difference started remarkably early. For upright faces, significant differences between fearful and neutral faces started 120 ms after stimulus onset (Fig. 1, top panel). When faces were presented upside-down (Fig. 1, bottom), the onset of this enhanced positivity for fearful as compared to neutral faces was delayed. The time course and scalp topography of these emotional expression effects is further illustrated in Fig. 2, which shows ERP scalp distribution maps for mean difference amplitudes, obtained by subtracting ERP waveforms in response to neutral faces from ERPs triggered by fearful faces within six successive post-stimulus latency windows. In these maps, enhanced positivities for fearful relative to neutral faces are shown in red colours, while blue colours indicate that amplitude differences were small or absent. Emotional positivities for upright fearful faces (Fig. 2, top panel) consisted of an early frontocentrally distributed effect (triggered between 110 and 200 ms after stimulus onset), and a more broadly distributed effect that started at about 250 ms post-stimulus, and remained present throughout the 1000 ms analysis interval. Emotional expression effects triggered by upside-down faces were substantially delayed and also attenuated (Fig. 2, bottom panel). They only emerged beyond 150 ms post-stimulus, and disappeared beyond 700 ms.

These ERP results suggest that emotional facial expression is analysed rapidly and can affect cortical processing at very short latencies (i.e., within about 120 ms after stimulus onset; see also Ashley, Vuilleumier, & Swick, 2003; Vuilleumier & Pourtois, 2006, this issue, for similar findings). One might of course argue that the early ERP differences shown in Figs. 1 and 2 are not directly related to emotional facial expression processing, but could instead be caused by systematic differences in low-level visual features of fearful versus neutral faces. However, the fact that face inversion, which preserves all low-level visual features, but makes the recognition of facial expression more difficult, resulted in an attenuation and a delay of these emotional expression effects, makes this alternative interpretation unlikely. It appears much more plausible to assume that the impact of face inversion on the latency and amplitude of ERP emotional expression effects directly reflects the impairment of emotion detection and recognition produced by face inversion.

The scalp distribution maps shown in Fig. 2 suggest that the processing of emotional facial expression, as reflected by ERP waveforms, might consist of two distinct stages. The early frontocentrally distributed positivity for fearful as compared to neutral faces could be linked to an initial rapid detection of facial expression in prefrontal areas involved in the detection of emotionally significant stimuli. In line with this assumption, Kawasaki et al. (2001) have measured differential responses of single neurons in right ventral prefrontal cortex in response to emotionally neutral versus aversive stimuli at latencies comparable to the onset latencies of early emotional expression effects shown in Figs. 1 and 2. In contrast, the sustained and more broadly distributed positivity obtained for fearful faces beyond 250 ms post-stimulus might reflect subsequent higher level stages of emotional face processing, such as the conscious evaluation of emotional content. Similar sustained positive ERP deflections have been observed in previous ERP studies in response to emotionally salient non-face stimuli (Cuthbert, Schupp, Bradley, Birbaumer, & Lang, 2000; Diedrich, Naumann, Maier, & Becker, 1997; Keil et al., 2002), thus suggesting that these longer latency effects are not face-specific, but can also be elicited by other kinds of emotional material.

## 3 ERP correlates of emotional face processing: other emotional expressions

In our initial study (Eimer & Holmes, 2002), we only studied the effects of fear on ERP waveforms. Fearful faces were chosen because they are known to modulate neocortical regions via the amygdala (Amaral & Price, 1984; Vuilleumier, Armony, Driver, & Dolan, 2001; see also Graham, Devinsky, & LaBar, 2006, this issue). However, there is now substantial evidence for the existence of neural systems that are specialised for processing distinct emotions. For example, the amygdala appears to be specifically sensitive to facial expressions of fear, while insula and basal ganglia have been linked to the processing of facial expressions of disgust (see Adolphs, 2002, for a more detailed review). If the detection and analysis of different facial emotional expressions was mediated by distinct brain processes, this might also be reflected in systematic differences in the modulatory effects of these expressions on ERP waveforms.

**Fig. 3:**
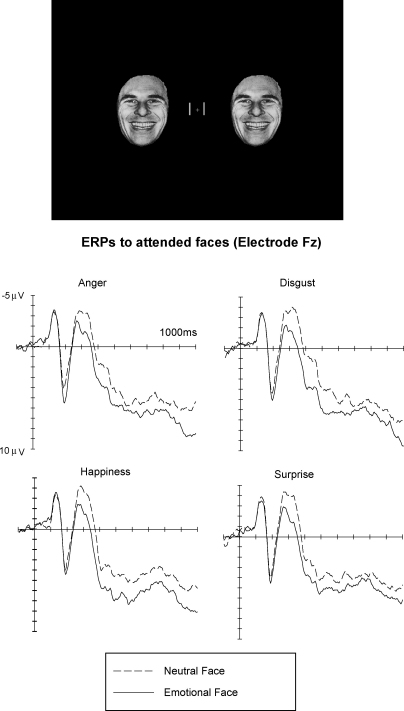
*Top:* Stimulus setup used in the Eimer et al. (2003) study. *Bottom:* Grand-averaged ERP waveforms elicited at midline electrode Fz in response to stimulus arrays containing neutral faces (dashed lines) or emotional faces (solid lines). ERPs are shown separately for blocks containing angry, disgusted, happy, and surprised faces. Data from Eimer et al. (2003).

To investigate whether different emotional facial expressions give rise to distinct modulations of ERP waveforms, or whether similar emotional expression effects can be observed for all basic emotional expressions, we conducted an experiment where ERP responses elicited by six basic emotional facial expressions (anger, disgust, fear, happiness, sadness, and surprise, see Ekman & Friesen, 1976) were directly compared (Eimer, Holmes, & McGlone, 2003). In each block, faces were either neutral or emotional (with emotional expression varied between blocks). In contrast to our first study (Eimer & Holmes, 2002), where faces were presented at fixation, we now delivered pairs of identical faces to the left and right of fixation together with a pair of vertical lines close to fixation (see Fig. 3, top). Participants had to decide on each trial whether the expression of the face pair was emotional or neutral. This procedure was chosen to investigate effects of spatial attention on the processing of emotional facial expression by comparing ERPs elicited when emotional and neutral faces were task-relevant to ERPs recorded in another part of the same experiment where faces were irrelevant, and attention was directed to the line stimuli instead (see Section 5).

Fig. 3 shows ERPs in response to task-relevant emotional and neutral faces elicited at frontal midline electrode Fz, separately for experimental blocks containing angry, disgusted, happy, and surprised faces. Emotional expression effects were very similar across different facial expressions. An enhanced positivity for emotional as compared to neutral faces started about 180 ms after stimulus onset, and a sustained emotional positivity was present throughout the 1000 ms recording epoch. These effects were elicited in a very similar fashion for all six emotions studied, and scalp topographies were statistically indistinguishable across emotions.^1^ It is notable that the onset of emotional expression effects shown in Fig. 3 was slightly delayed relative to the onset latencies observed in our earlier study (Eimer & Holmes, 2002), where frontal differences between ERPs to upright fearful and neutral faces were already present at 120 ms post-stimulus (see Figs. 1 and 2). This difference is most likely due to the fact that face stimuli were now presented peripherally, rather than centrally, and were accompanied by potentially competing non-face stimuli (vertical lines) close to fixation (see Fig. 3, top).

The similarity in the onset latencies of ERP emotional expression effects for all six basic emotional expressions suggests that emotionally relevant information delivered by facial expression is available to neocortical processes within less then 200 ms after stimulus onset, and at approximately the same time for all basic emotional expressions. The observation that all six emotional facial expressions were also very similar in terms of the magnitude and duration of their effects on ERP waveforms relative to neutral faces, which was confirmed by statistical analyses (see Eimer et al., 2003, for details), appears to be at odds with the hypothesis that distinct neural sub-systems specialise in the processing of specific emotions (Adolphs, 2002). If this was the case, one might have expected systematic differences between ERP emotional expression effects elicited by different facial expressions. While many neuroimaging studies have shown emotion-specific activation patterns of brain regions such as the amygdala or insula (Adolphs, Tranel, & Damasio, 2003; Breiter et al., 1996; Calder, Keane, Manes, Antoun, & Young, 2000; Calder, Lawrence, & Young, 2001; Morris et al., 1996; Phillips et al., 1997, 1998; Sprengelmeyer, Rausch, Eysel, & Przuntek, 1998; Whalen et al., 2001), fMRI measures are insensitive to the time scale of such effects. Thus, it is possible that some of these effects occur substantially later than the early ERP modulations found in our studies. Initial evidence for this assumption comes from recent intracranial recordings of ERPs to facial expressions (Krolak-Salmon, Henaff, Vighetto, Betrand, & Maugiere, 2004). In this study, fear-specific amygdala responses were observed at latencies of 200 ms and beyond, that is, well after the early fear-specific ERP modulations described above. In addition, relatively few fMRI studies to date have pointed to differential activation within surface neocortical structures (where effects of emotional expression on ERPs are likely to be generated). It is thus possible that early stages in the processing of emotionally relevant information, subserved by limbic structures or the basal ganglia, and neocortical emotional processing stages differ in terms of their specificity.

It has to be acknowledged that these tentative interpretations are primarily based on the results of a single ERP study, where systematic differences in ERP responses to specific facial expressions were conspicuously absent (Eimer, Holmes, & McGlone, 2003). One could argue that this negative result may be primarily due to methodological factors. For example, the same neutral face stimuli were presented in all blocks, whereas the expression of emotional faces changed across blocks. This could have resulted in different rates of habituation for emotional versus neutral faces, regardless of emotional expression (see Carretie, Hinojosa, & Mercado, 2003, for ERP correlates of habituation to emotional stimuli). However, the fact that the emotional expression effects observed in this experiment were qualitatively very similar to the effects observed in our other studies where emotional and neutral faces were equiprobable makes this interpretation somewhat unlikely. Alternatively, it could be argued that subtle topographical differences in the impact of emotional facial expression on electrical brain responses may only be detectable when combining high-density EEG or MEG recordings and source localization analyses. In fact, several recent studies have reported systematically different ERP modulations in response to different emotional expressions (e.g., Batty & Taylor, 2003; Pourtois, Grandjean, Sander, & Vuilleumier, 2004). In light of these results, it is clear that the question whether or not different emotional facial expressions give rise to specific ERP effects is far from settled, and that further systematic studies of electrophysiological correlates of emotional face processing are needed to find a definitive answer.

## 4 Structural encoding and facial expression processing are independent and parallel processes

Evidence from neuropsychology strongly suggests that the processing of facial identity and facial expression are subserved by dissociable neural substrates, in line with the model of face processing proposed by Bruce and Young (1986). Focal damage to selective brain regions can leave some patients with a deficiency in recognising faces, and yet spare the ability to read facial expressions of emotion (Etcoff, 1984; Posamentier & Abdi, 2003; Tranel & Damasio, 1988; Young, Newcombe, de Haan, Small, & Hay, 1993). On the other hand, some patients are impaired in their ability to read emotional cues from faces, but have no difficulty in identifying people (Hornak, Rolls, & Wade, 1996). Informed by such double dissociations, models of face processing such as those proposed by Bruce and Young (1986) and by Haxby et al. (2000) have incorporated the assumption that the detection and analysis of facial emotional expression and the structural encoding of facial features for face recognition are implemented by separable, independent, and parallel processes.

**Fig. 4:**
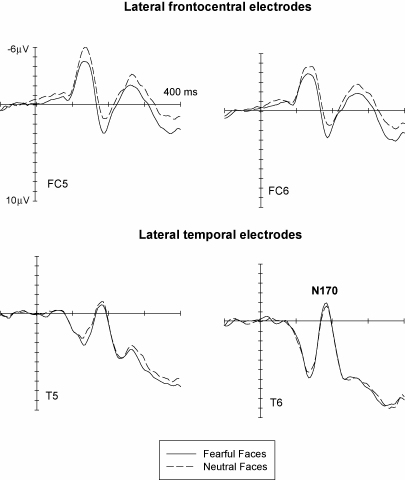
Grand-averaged ERPs in response to fearful faces (solid lines) and neutral faces (dashed lines) at lateral frontocentral electrodes (FC5/6, top panel) and at lateral temporal electrodes (T5/6, bottom panel). Data from Eimer and Holmes (2002).

The ERP studies reviewed so far (Eimer & Holmes, 2002; Eimer et al., 2003) have yielded further findings that are relevant to this hypothesis that the perceptual structural encoding and subsequent recognition of faces and the detection and analysis of emotional facial expression are independent. Fig. 4 (bottom) shows the face-specific N170 component, as observed in our initial study (Eimer & Holmes, 2002) at lateral temporal electrodes T5/6 in response to fearful and neutral faces. Numerous ERP studies have demonstrated that this component is triggered reliably by faces at lateral posterior electrodes at about 170 ms post-stimulus, but not by other types of objects. The N170 component is assumed to reflect the pre-categorical perceptual encoding of faces in face-specific ventral visual areas, which provides structural representations that are utilized by subsequent face recognition stages (Bentin, Allison, Puce, Perez, & McCarthy, 1996; Eimer, 1998, 2000).

As can be seen from Fig. 4 (bottom), the N170 was not at all sensitive to facial emotional expression, as no systematic differences in N170 amplitudes or latencies were observed for fearful as compared to neutral faces. This was confirmed in another study (Eimer et al., 2003), where the N170 component was found to be unaffected by the presence versus absence of any of the six basic emotional expressions. These findings suggest that the structural encoding of faces, as reflected by the N170, is insensitive to information derived from emotional facial expression.^2^ It is interesting to note in this context that several fMRI studies (Morris et al., 1998; Vuilleumier et al., 2001) have found that activity within face-specific fusiform areas is modulated by emotional facial expression. The fact that the N170 is insensitive to emotional expression could indicate that this component reflects face processing at some other anatomical stage than fusiform gyrus. In fact, there is now evidence from source localization studies (e.g., Itier & Taylor, 2004) that the N170 may at least in part be generated in more lateral temporal regions such as the superior temporal sulcus. Alternatively, the N170 might be triggered during an early expression-independent face processing stage located within the fusiform face area, which is too transient to be picked up reliably with hemodynamic measures, but is followed at longer latencies by a more sustained selective processing of facial expression in fusiform gyrus. In this context, it is interesting to note that intracranial recordings from ventral and lateral occipitotemporal and temporal regions have identified a sequence of face-specific potentials, starting with the N200 (Allison, Puce, Spencer, & McCarthy, 1999), and have found that only longer latency components are affected by affect, familiarity, and priming (Puce, Allison, & McCarthy, 1999).

To illustrate that structural encoding and emotional face processing, as reflected by ERP measures, represent separable stages, Fig. 4 contrasts ERPs observed in our initial study (Eimer & Holmes, 2002) in response to fearful and neutral faces at lateral posterior electrodes (where the N170 component was found to be insensitive to facial expression, bottom panel) with ERPs recorded at the same time at lateral frontocentral electrodes, where emotional expression effects were clearly present at and even before 170 ms post-stimulus. This pattern of results, which was replicated in our other studies (Eimer et al., 2003; Holmes, Vuilleumier, & Eimer, 2003), strongly suggests that the rapid detection of emotional facial expression within anterior brain regions occurs independently and in parallel with the construction of a detailed perceptual representation of faces within face-specific posterior ventral areas. They provide new evidence for the existence of functionally separable and parallel brain processes involved in the structural encoding of faces and in the processing of emotional facial expression (see also Parry, Young, Saul, & Moss, 1991, for further neuropsychological evidence for dissociable impairments in face processing after brain injury).

## 5 The impact of attention on the processing of emotional facial expression

It is often assumed that affectively salient stimuli such as emotional facial expressions are detected pre-attentively, and attract attention automatically (see Palermo & Rhodes, 2006, this issue, for detailed discussion). Strong attentional biases towards emotional stimuli have indeed been found in behavioural studies for visual search tasks (Eastwood, Smilek, & Merikle, 2001; Fox et al., 2000; Hansen & Hansen, 1988; Öhman, Flykt, & Esteves, 2001; Öhman, Lundqvist, & Esteves, 2001), or dot probe detection tasks (Holmes, Green, & Vuilleumier, 2005; Holmes, Richards, & Green, 2006; Mogg & Bradley, 1999; Mogg et al., 2000). However, recent brain imaging studies have yielded conflicting findings with respect to the influence of attention on the processing of affective material. On the one hand, amygdala responses to fearful faces in humans appear to be unaffected by spatial attention (Vuilleumier et al., 2001), and amygdala activations triggered by highly arousing emotional scenes are not modulated by a secondary task (Lane, Chua, & Dolan, 1999). In addition, neglect and extinction patients are more likely to detect emotionally significant relative to neutral pictures when these are presented in the affected visual hemifield (Vuilleumier & Schwartz, 2001a, 2001b). These results suggest that emotional stimuli can capture attention automatically. However, other recent fMRI studies have found attentional modulations of amygdala responses to fearful or happy facial expressions (Pessoa, Kastner, & Ungerleider, 2002; Pessoa, McKenna, Gutierrez, & Ungerleider, 2002), as well as increased responses to attended versus unattended fearful faces in the anterior temporal pole and anterior cingulate gyrus (Vuilleumier et al., 2001).

**Fig. 5:**
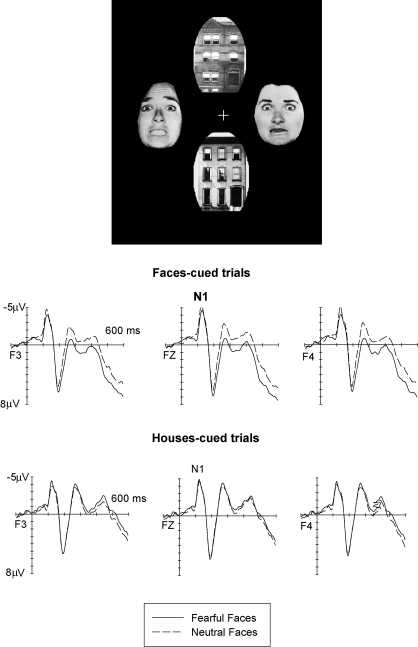
*Top:* Stimulus setup used in the Holmes et al. (2003) study. *Bottom:* Grand-averaged ERP waveforms elicited at midline electrode Fz at lateral frontal electrodes F3/4 in response to stimulus arrays containing fearful faces (solid lines) or neutral faces (dashed lines), shown separately for trials where faces were presented at attended locations (faces-cued trials), and for trials where houses were presented at attended locations and faces were unattended (houses-cued trials). Data from Holmes et al. (2003).

We investigated the role of attention in the processing of emotional facial expression by studying whether and how directing attention towards or away from emotional faces would affect any emotion-induced modulations of ERP waveforms. In one study (Holmes et al., 2003), we recorded ERPs in response to stimulus arrays that consisted of two faces and two houses arranged in horizontal and vertical pairs (see Fig. 5, top). The location of the face and house pairs (vertical versus horizontal) and the emotional expression of the face pair (fearful versus neutral) was varied randomly across trials. Participants had to direct their attention either to the two vertical or to the two horizontal locations in order to detect infrequent target events (two identical faces or two identical houses at these attended locations). Stimuli at the two other locations could be ignored. Task-relevant locations changed randomly across trials, and were indicated by two boxes presented at the start of each trial.

Fig. 5 shows ERPs triggered in response to stimulus arrays containing either fearful or neutral faces, separately for trials where faces were presented at cued (attended) positions (faces-cued trials), and for trials where houses were presented at cued locations, while faces were task-irrelevant and could therefore be ignored (houses-cued trials). When faces were attended, the results obtained in our previous studies (Eimer & Holmes, 2002; Eimer et al., 2003) were basically replicated. Fearful faces triggered an enhanced positivity relative to neutral faces, with an early frontocentral effect and a subsequent more broadly distributed sustained emotional positivity. Surprisingly, these emotional expression effects were completely eliminated on trials where faces were presented at unattended locations (Fig. 5, houses-cued trials). Here, no statistically significant modulations of ERPs in response to arrays containing fearful versus neutral faces were obtained at all. This finding that emotion-specific ERP modulations are strongly dependent on spatial attention challenges the hypothesis that the detection and processing of emotional facial expression occurs pre-attentively, and suggests that the processes reflected by ERP modulations sensitive to emotional facial expression are gated by spatial attention.

**Fig. 6:**
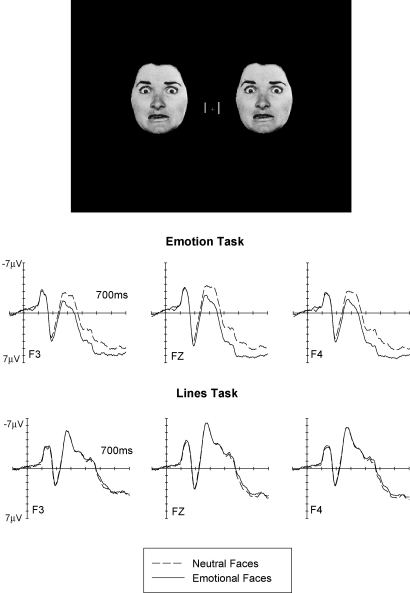
*Top:* Stimulus setup used in the Eimer et al. (2003) study. *Bottom:* Grand-averaged ERP waveforms elicited at midline electrode Fz at lateral frontal electrodes F3/4 in response to stimulus arrays containing emotional faces (solid lines) or neutral faces (dashed lines), shown separately for blocks where faces were task-relevant and therefore attended (Emotion Task), and for blocks where lines were task-relevant and faces were unattended (Lines Task). Data from Eimer et al. (2003).

In order to confirm and extend this surprising finding that ERP modulations triggered by emotional faces are contingent upon faces being attended, we investigated the impact of directing attention towards or away from faces for all six basic facial expressions. This was done in the study (Eimer et al., 2003) already introduced in Section 3, where stimulus arrays consisted of pairs of identical peripheral faces that were presented together with a central pair of vertical lines (Fig. 6, top). Facial expression was either neutral, or angry, disgusted, fearful, happy, sad, or surprised, with emotional expression varied across blocks. To assess the impact of spatial attention on the processing of emotional facial expression, we compared two different tasks where attention was either directed towards or away from the face stimuli. In the Emotion task described in Section 3, faces were attended, since participants were instructed to judge their emotional expression. In the Lines task, attention was directed away from these faces, as participants had to report whether the two vertical lines were identical or differed in length.

Fig. 6 shows ERPs triggered at frontal recording sites in response to emotional faces (collapsed across all six emotional facial expressions) and neutral faces in the Emotion task where faces were attended and in the Lines task where attention was directed away from these faces. In the Emotion task, emotional faces elicited an enhanced positivity starting at about 160 ms post-stimulus (see also Section 3 and Fig. 3), similar to the results from our other ERP studies (Eimer & Holmes, 2002; Holmes et al., 2003). In marked contrast, emotional expression effects were completely eliminated in the Lines task (Fig. 6, bottom). When attention was directed away from face stimuli towards the perceptually difficult central line discrimination task, the presence of emotional versus neutral faces had no effect whatsoever on ERP waveforms, and this was the case for each of the six basic emotions studied (see Eimer et al., 2003, for more details).

**Fig. 7:**
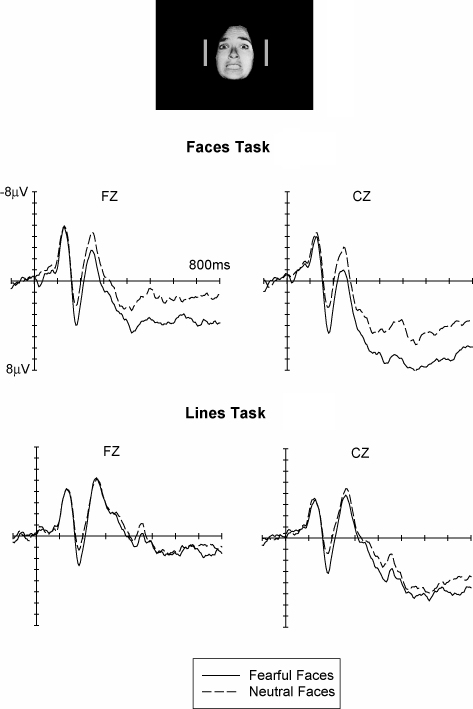
*Top:* Stimulus setup used in our study of the impact of selective attention on the processing of foveal emotional faces. *Bottom:* Grand-averaged ERP waveforms elicited at midline electrodes Fz and Cz in response to stimulus arrays containing emotional faces (solid lines) or neutral faces (dashed lines), shown separately for blocks where faces were task-relevant and therefore attended (Emotion Task), and for blocks where lines were task-relevant and faces were unattended (Lines Task). Data from Holmes et al. (2006a).

In the light of these findings that emotional expression effects on ERP waveforms were completely eliminated when attention was directed away from laterally presented emotional faces to other task-relevant locations, we next wanted to find out whether attention would have a similar impact on emotional face processing even when faces are located at fixation. With emotional faces presented within foveal vision, we expected to find little if any modulatory effects of attention on emotional expression effects. This was tested in another ERP study (Holmes, Kiss, & Eimer, 2006), where single faces (either fearful or neutral) were presented foveally at fixation, and were flanked by a pair of vertical lines that were either identical or different in length (see Fig. 7, top). In one half of the experiment, faces were attended, since participants had to detect immediate repetitions of the same face stimulus on successive trials. In the other experimental half, they had to detect immediate repetitions of an identical line pair on successive trials, and faces could be entirely ignored.

Fig. 7 shows grand-averaged ERP waveforms obtained from ten participants in response to fearful and neutral faces at midline electrodes Fz and Cz when attention was directed to central faces (Faces task, top) or to lateral lines (Lines task, bottom). Similar to the results obtained before, fearful faces again triggered an enhanced positivity relative to neutral faces when faces were attended, and this effect started at about 150 ms post-stimulus. In contrast to our findings for lateral faces, these emotional expression effects were not entirely eliminated when attention was directed to the lateral lines. As can be seen from Fig. 7, the early phase of this effect triggered between 150 and 200 ms post-stimulus was clearly preserved even when instructions required participants to ignore foveal faces, and statistical analyses revealed that a significantly enhanced positivity to fearful faces was present regardless of attention instructions. In contrast, longer latency emotional expression effects, which were present when faces were attended, were strongly attenuated and failed to reach statistical significance in the Lines task where participants were instructed to ignore faces, and to judge lateral lines instead.

These observations suggest that there are important differences in the impact of attention on cortical stages of emotional expression processing of foveal and peripheral faces. When faces are presented at fixation, an initial rapid detection of their emotional expression (as reflected by early emotional expression effects) takes place irrespective of attentional task instructions. In contrast, no ERP evidence for emotional expression processing can be found when peripherally presented faces are unattended. However, and equally importantly, it appears that subsequent stages in emotional face processing are under full strategic control, and are therefore only activated when attentional task instructions require observers to process these faces, irrespective of whether they are presented foveally or in the periphery of the visual field.

The ERP studies presented in this section have demonstrated that emotion-specific ERP modulations are strongly dependent on spatial attention. The observation that both short-latency as well as sustained longer latency ERP emotional expression effects are completely eliminated when emotional faces are presented at lateral positions and outside of the current focus of attention is clearly at odds with the hypothesis that emotional facial expression is always fully processed, regardless of other current task demands. It should be noted that our observation that ERP modulations sensitive to emotional facial expression are gated by spatial attention contrasts markedly with fMRI results showing that amygdala responses to fearful faces and other emotionally salient stimuli are unaffected by attention (Anderson, Christoff, Panitz, De Rosa, & Gabrieli, 2003; Lane et al., 1999; Vuilleumier et al., 2001; see also Öhman & Mineka, 2001, for a description of a module for fear elicitation that is cognitively impenetrable and activated automatically). Recent functional imaging studies have even demonstrated fear-specific amygdala activation during binocular suppression (Pasley, Mayes, & Schultz, 2004; Williams, Morris, McGlone, Abbot, & Mattingley, 2004), which was interpreted as evidence for a direct subcortical pathway to the amygdala.

The striking differences between these results and the ERP findings discussed in this section suggest that frontal neocortical and limbic systems involved in the analysis of emotional events might differ with respect to their functional properties. Rapid amygdala responses may be triggered by attended as well as unattended emotional stimuli (although these responses may still be modulated by selective attention; see Pessoa et al., 2002a, 2002b). In contrast, neocortical stages of emotional processing (as reflected by the ERP effects discussed here) appear to be much more dependent on focal attention. At these stages, emotionally salient events such as emotional faces may not have a unique status with respect to their immunity to attentional capacity limitations. When attention is already actively engaged elsewhere, the cortical processing of emotional facial expressions may therefore be similarly impaired as the processing of other types of perceptual objects.

## 6 The impact of spatial frequency on emotional expression analysis

One distinctive feature of brain structures sensitive to emotionally relevant information such as the amygdala, superior colliculus and pulvinar is that they appear to be preferentially activated by low spatial frequency (LSF), but not high spatial frequency (HSF) representations of fearful faces (Vuilleumier, Armony, Driver, & Dolan, 2003; Winston, Vuilleumier, & Dolan, 2004). This observation is consistent with anatomical evidence that these brain areas receive substantial magnocellular inputs (Berson, 1988; Leventhal, Rodieck, & Dreher, 1985; Schiller, Malpeli, & Schein, 1979), possibly as part of a phylogenetically old route specialised for the rapid processing of fear-related stimuli (Le Doux, 1996; Morris, Öhman, & Dolan, 1999). In contrast, parvocellular channels, which are more responsive to HSF stimuli, provide input to ventral visual cortex, but not subcortical areas, and are crucial for the detailed processing of shape and colour on which object and face recognition are based (Breitmeyer, 1992; Livingstone & Hubel, 1988). A recent behavioural study conducted in our lab (Holmes et al., 2005a) has also revealed that rapid attentional responses to fearful versus neutral faces are driven by LSF rather than HSF information, consistent with the suggested role of amygdala in the mediation of attention towards emotional stimuli (Morris et al., 1998; Vuilleumier et al., 2001).

**Fig. 8:**
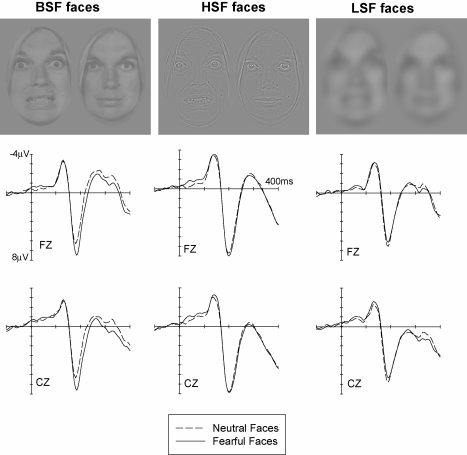
Grand-averaged ERP waveforms elicited at midline electrodes Fz and Cz in response to fearful faces (solid lines) and neutral faces (dashed lines), shown separately for broadband (BSF) faces (left), high spatial frequency (HSF) faces (middle), and low spatial frequency (LSF) faces (right). Data from Holmes et al. (2005b).

Given the assumed preference of amygdala and connected structures for LSF information, driven by magnocellular afferents, we have recently conducted a study (Holmes, Winston, & Eimer, 2005) to investigate whether effects of emotional facial expression on ERP waveforms would show a similar selectivity. ERPs were recorded while participants viewed photographs of centrally presented faces with fearful or neutral expressions, houses, or chairs. Their task was to detect and respond to infrequently presented target stimuli (chairs). Fig. 8 (top) shows examples of the different types of face stimuli used in this study. Broad spatial frequency (BSF) stimuli were unfiltered, LSF stimuli were low-pass filtered to retain only frequencies below 6 cycles per image (2 cycles/degree of visual angle), while HSF stimuli were high-pass filtered to retain only frequencies above 26 cycles per image (4 cycles/degree of visual angle). To preclude confounds due to differences between these stimulus categories in terms of their brightness or contrast, all stimuli were normalized for their luminance and average spectral energy.

Fig. 8 shows ERPs elicited at midline electrodes Fz and Cz in the first 400 ms after stimulus onset in response to fearful and neutral faces, separately for BSF faces (left panel), HSF faces (middle panel), and LSF faces (right panel). As expected, emotional expression had a strong effect on ERPs elicited by BSF faces. Confirming previous findings, an enhanced and statistically reliable positivity for fearful relative to neutral faces started at about 150 ms post-stimulus. In contrast, there was no clear evidence for differential ERP responses to fearful versus neutral faces in response to HSF and LSF faces (see Fig. 8, middle and right panels). The absence of systematic emotional expression effects for these filtered faces was also confirmed by statistical analyses. If LSF cues were more important than HSF cues in producing ERP modulations to fearful facial expressions, one would have expected to find stronger emotional expression effects for LSF as compared to HSF faces, with effects for LSF faces possibly even similar in magnitude to expression effects observed for unfiltered BSF faces. In fact, ERP responses to LSF faces were entirely unaffected by emotional expression. Given that emotional processes in amygdala and related brain regions have been found to be selectively driven by LSF signals (Vuilleumier et al., 2003; Winston et al., 2004), the absence of any reliable ERP effects of facial expression for LSF faces again suggests that these ERP responses do not directly reflect activity in these regions, but are instead produced by functionally distinct neocortical brain systems.

## 7 Conclusions

In the studies reviewed in this paper, ERPs were measured in response to emotional and neutral faces. We investigated the onset, time course, and topographic distribution of brain responses to emotional faces in order to learn more about functional properties of emotional face processing. These studies have revealed robust and replicable differential ERP responses to emotional versus neutral faces. In all experiments reviewed in this paper, emotional faces triggered an increased positivity relative to neutral faces. The onset of this emotional expression effect was remarkably early, ranging from 120 ms post-stimulus when faces were presented at fixation and no competing non-face stimuli were simultaneously present (Eimer & Holmes, 2002) to 180 ms post-stimulus when emotional faces were presented at peripheral locations together with other stimuli close to fixation (Eimer et al., 2003). While we assume that factors such as the retinal position of faces and the presence versus absence of potentially competing non-face stimuli in the display are largely responsible for such onset latency differences, the possibility remains that other cognitive factors might determine the onset of these effects. It is unlikely that the presence of an explicit emotion task plays an important role, given that very early emotional expression effects were observed under conditions where participants had to detect the immediate repetitions of face as well as non-face stimuli, and emotional expression was entirely irrelevant (Eimer & Holmes, 2002). The intriguing possibility that the onset of these effects might also vary as a function of participants’ trait or state anxiety (see Bishop, Duncan, & Lawrence, 2004; Fox, 2002, for recent behavioural and neuroimaging evidence for an impact of anxiety on emotional processing) will be investigated in future studies.

The initial phase of the emotional expression effects observed in the studies reviewed here showed a frontocentral scalp distribution, while its later phase beyond 250 ms post-stimulus was more broadly distributed (see Fig. 2), thus strongly suggesting that different neural generators are activated during early and later stages of this emotional positivity. While similar broadly distributed longer latency positivities have been reported in previous ERP studies for emotionally salient non-face stimuli (Cuthbert et al., 2000; Diedrich et al., 1997; Keil et al., 2002), differential ERP modulations at latencies below 200 ms have not been found with other types of emotional material, thus suggesting that such rapid effects might be specific for emotional faces (see Ashley et al., 2003, for similar early ERP effects of emotional facial expression).

The short onset latency of emotional expression effects observed in the ERP studies reviewed in this paper might suggest that the early phase of these effects reflects the rapid pre-attentive automatic assessment of the emotional content of faces, as implemented by structures such as the amygdala. One obvious objection against this hypothesis is that it is highly unlikely that the effects of emotional expression on ERP waveforms reviewed in this paper are generated in the amygdala, given its deep position and its electrically closed nuclear structure of clustered neurons (unlike the regular alignment of neurons in layers of the neocortex). Of course, even though amygdala activations themselves are unlikely to result in measurable ERP responses at the scalp surface, rapid automatic amygdala activations triggered by emotional faces might still be relayed directly to neocortical areas (Morris et al., 1998), where they could be picked up via ERPs. It is conceivable that the early emotional expression effects observed in our ERP studies reflect activity within a neural network involved in the rapid automatic classification of emotional faces, which includes limbic structures as well as interconnected neocortical regions. However, several other findings from our ERP studies provide strong evidence against such a hypothesis. First, we found that ERP emotional expression effects were triggered at comparable latencies, and showed similar amplitudes and scalp topographies for all six basic emotions (Eimer et al., 2003). Although clearly preliminary (see the cautionary comments in Section 3), this finding contrasts markedly with the emotion-specificity of the neural structures assumed to be involved in the rapid automatic evaluation of emotional content. Several studies have found a disproportionate activation of the amygdala in response to facial expressions of fear (Breiter et al., 1996; Morris et al., 1996; Phillips et al., 1998; Whalen et al., 2001). Insula and basal ganglia seem to be particularly involved in processing facial expressions of disgust (Adolphs et al., 2003; Calder et al., 2000, 2001; Phillips et al., 1997, 1998; Sprengelmeyer et al., 1998), and prefrontal areas appear to be specifically implicated in the recognition of angry faces (Blair, Morris, Frith, Perrett, & Dolan, 1999; Harmer, Thilo, Rothwell, & Goodwin, 2001). Second, we found that ERP responses to emotional faces are not selectively driven by low spatial frequency information (Holmes et al., 2005b). Again, this observation contrasts with previous observations for structures subserving the automatic classification of emotional input, such as the amygdala, which has been found to be preferentially activated by LSF signals (Vuilleumier et al., 2003; Winston et al., 2004). And finally, fear-specific amygdala activation appears to start only at latencies of 200 ms (Krolak-Salmon et al., 2004), and thus considerably later than the early emotional expression effects observed in our studies. This observation casts further doubt on the hypothesis that rapid amygdala signals are responsible for these ERP effects.

The most important evidence against the hypothesis that ERP modulations sensitive to emotional facial expression are generated during the initial automatic classification of emotional content comes from our finding that spatial attention had a strong modulatory effect on these modulations. When faces were presented foveally, early emotional expression effects (but not longer latency effects) were triggered independently of the current focus of attention. However, and most importantly, even these early effects were completely eliminated when attention was directed away from the location of peripheral emotional faces towards another perceptual task (Eimer et al., 2003; Holmes et al., 2003). This observation is clearly at odds with the idea that early emotional expression effects reflect the pre-attentive registration of facial expression. Recent fMRI evidence suggests that amygdala responses to fearful faces are unaffected by attention (Lane et al., 1999; Vuilleumier et al., 2001), indicating that the amygdala is part of a network involved in the pre-attentive automatic detection of emotional content. The strong dependence of ERP emotional expression effects on spatial attention demonstrated in our studies implies that the processes responsible for the generation of these effects are functionally distinct from this pre-attentive detection network. One could speculate that this remarkable effect of attention on emotional expression processing, as reflected by the absence of any differential ERP effects for unattended emotional faces, might be mediated by control structures in orbitofrontal cortex. Orbitofrontal regulatory processes can suppress responses to emotional stimuli in the amygdala as well as in higher-order emotion areas (Davidson, 2002; Freedman, Black, Ebert, & Binns, 1998; Rolls, 1996). Orbitofrontal cortex has also been implicated in monitoring and restricting affective impulses through feedback mechanisms (Rolls, 1999; see also Rolls, 2006, this issue), and has been posited as a key structure in the reallocation of attention when emotional faces are task irrelevant (Vuilleumier et al., 2001).

It is conceivable that the absence of early emotional expression effects in response to unattended peripheral emotional faces found in our previous studies (Eimer et al., 2003; Holmes et al., 2003) may be due to the fact that observers were unable to identify facial expressions when attention was directed elsewhere. Although face identification performance was excellent when peripheral faces were attended, the possibility that observers are unaware of emotional expressions when attention is diverted has not yet been explicitly addressed. Links between attention, early emotional expression effects, and the presence versus absence of conscious awareness of facial expressions will need to be systematically investigated in future studies.

It should also be stressed that amygdala activations in response to emotional stimuli do not always and exclusively represent a rapid and automatic classification of these stimuli. Attentional modulations of amygdala responses to fearful or happy facial expressions have in fact been observed (Pessoa et al., 2002a, 2002b), and modulations of amygdala activation were also found as a function of top–down control processes involved in the intentional regulation or reinterpretation of affective information (Ochsner, Bunge, Gross, & Gabrielli, 2002; Schaefer et al., 2002). These findings suggest that the amygdala may also play an important role during later processing stages where the processing of emotional information is under attentional control and conscious representations of emotional content are generated.

Overall, our findings that ERP modulations in response to emotional faces were triggered in a very similar fashion for all basic facial expressions, were not selectively driven by low spatial frequency information, but were strongly modulated by attention all suggest that these effects are elicited during stages in the processing of emotional information that are located beyond the initial rapid and automatic classification of emotional content. The outcome of an initial rapid and automatic appraisal of emotional stimuli will be fed into higher necortical stages of emotional processing, where the evaluation of emotional material is likely to continue in parallel with ongoing emotion evaluation in amygdala and related brain circuits. We suggest that emotional expression effects, as reflected by ERP waveforms in response to emotional faces, reflect processes at this later neocortical stage in the processing of emotional information.

At present, it would be premature to speculate in more detail about specific cortical regions where the ERP effects reviewed in this paper might be generated, although regions such as anterior cingulate, somatosensory cortex, or medial prefrontal cortical areas seem plausible candidates. For example, Vuilleumier, Armony, Driver, and Dolan (2001) have identified a dorsal region of the anterior cingulate, which showed greater activation when emotional faces were attended, analogous to our finding that ERP effects of emotional expression are strongly affected by attention. The dorsal subdivision of the anterior cingulate has been implied in functions such as attentional control, error and response conflict monitoring (Bush, Luu, & Posner, 2000; Paus, Koski, Caramanos, & Westbury, 1998; Posner & Rothbart, 1998), but also as a potential locus of emotional awareness (Lane, Reiman, Axelrod, Holmes, & Schwartz, 1998), consistent with evidence that this region participates directly in the affective component of personally experienced (Rainville, Duncan, Price, Carrier, & Bushnell, 1997) or observed (Hutchison, Davis, Lozano, Tasker, & Dostrovsky, 1999) pain.

The analysis of emotional facial expression is based on a complex neural network, and includes both a rapid, obligatory, and pre-attentive classification of emotional content (implemented within the amygdala, orbitofrontal cortex, and ventral striatum), and the subsequent in-depth analysis of emotional faces in higher order necortical emotion areas (including somatosensory cortex, anterior cingulate, and medial prefrontal cortices). In spite of the fact that ERP effects of emotional facial expression are triggered at very short latencies, they are likely to reflect processes that form part of the second, higher level and attention-dependent emotional processing system, where representations of emotional content are generated in a strategic and task-dependent fashion for the adaptive intentional control of behaviour. Further studies will have to investigate to what extent effects of emotional facial expression on ERP waveforms, such as reviewed here, might represent the initial stages of higher level emotional processes, which may not just be involved in the analysis of emotionally relevant sensory stimuli, but perhaps also in the representation of subjective emotional states.

## Footnotes

1 Emotional expression effects for fearful and disgusted faces, which are not shown in Fig. 3, were also similar to and statistically indistinguishable from the effects observed for the other emotions (see Eimer et al., 2003 for more details).

2 It should be noted that in contrast to the findings reported here, some other studies have reported effects of emotional facial expression on early posterior ERP component, such as the P1 and N170 (e.g., Batty & Taylor, 2003), or even the earlier C1 component which is assumed to be generated in primary visual cortex (Pourtois et al., 2004, 2005), thus suggesting that under certain experimental conditions, emotional facial expression may be able to modulate early stages of perceptual processing in sensory-specific visual areas.
